# Supplementary material for: Electronic data capture for large scale typhoid surveillance, household contact tracing, and health utilisation survey: Strategic Typhoid Alliance across Africa and Asia
Source: Wellcome Open Res. 2020 Dec 1;5:66. Originally published 2020 Apr 9. [Version 2] doi: 10.12688/wellcomeopenres.15811.2 (PMC7471626; doi:10.12688/wellcomeopenres.15811.2)
Supplement: Peer review history

# Peer review: Reviewer response for version 2

**DOI:** 10.21956/wellcomeopenres.18126.r41690

**Published:** 2020-12-07

**Authors:** Sylvester Maleghemi

**Affiliations:** 1 World Health Organization, Port Harcourt, Nigeria

Thank you for the revised version, more discussion could have been spared on the discrepancy in the completeness as a 20% difference is higher and affect the validity of this study.

 What were the main issues so that if we need to replicate the EDCs use in other countries it is avoided?

Are the conclusions about the tool and its performance adequately supported by the findings presented in the article?

Partly

Is the rationale for developing the new software tool clearly explained?

Partly

Is the description of the software tool technically sound?

Yes

Are sufficient details of the code, methods and analysis (if applicable) provided to allow replication of the software development and its use by others?

Yes

Is sufficient information provided to allow interpretation of the expected output datasets and any results generated using the tool?

Yes

Reviewer Expertise:

My areas of research include Immunology, use of emobile technology for health, data management, clinical trials, research and International Health regulation.

I confirm that I have read this submission and believe that I have an appropriate level of expertise to confirm that it is of an acceptable scientific standard.

# Peer review: Reviewer response for version 1

**DOI:** 10.21956/wellcomeopenres.17339.r39896

**Published:** 2020-09-03

**Authors:** Gagandeep Kang

**Affiliations:** 1 Division of Gastrointestinal Sciences, Christian Medical College, Vellore, India

- Electronic data capture is essential for clinical research. Data quality has been an issue, particularly where field staff with limited education are responsible for data capture. The authors have described how they built an eCRF using Open Data Kit and MySQL and deployed it for capturing data from over 300,000 individuals from 80,000 households.
- The group collected baseline data from households for a census with an error rate of less than 50 per 10,000 in all three sites. Expectedly, text fields had more errors. While low, the error rate in Nepal was almost double other sites. It would be useful to know whether there were specific fields that were problematic in one location and not others? Or whether the errors were distributed evenly across field staff?
- A key issue with electronic data capture is the availability of internet access and the group has addressed the combination with offline collection and twice daily uploading. It would help to have clarity on whether there are country-level regulatory/legal issues with the data being hosted with the Oxford Vaccine Group?
- While this was not included in the scope of the paper, the authors’ comments on whether baseline census information data collection accuracy was comparable to data collected during follow-up or plans for future analysis would be helpful.

Are the conclusions about the tool and its performance adequately supported by the findings presented in the article?

Yes

Is the rationale for developing the new software tool clearly explained?

Yes

Is the description of the software tool technically sound?

Yes

Are sufficient details of the code, methods and analysis (if applicable) provided to allow replication of the software development and its use by others?

Yes

Is sufficient information provided to allow interpretation of the expected output datasets and any results generated using the tool?

Yes

Reviewer Expertise:

Public health

I confirm that I have read this submission and believe that I have an appropriate level of expertise to confirm that it is of an acceptable scientific standard.

# Response

**Published:** 2020-11-23

**Authors:** Deus Thindwa

**Affiliations:** Malawi Liverpool Wellcome Trust, Malawi

*Electronic data capture is essential for clinical research. Data quality has been an issue, particularly where field staff with limited education are responsible for data capture. The authors have described how they built an eCRF using Open Data Kit and MySQL and deployed it for capturing data from over 300,000 individuals from 80,000 households.*

**Response**: Thank you.

*The group collected baseline data from households for a census with an error rate of less than 50 per 10,000 in all three sites. Expectedly, text fields had more errors. While low, the error rate in Nepal was almost double other sites. It would be useful to know whether there were specific fields that were problematic in one location and not others? Or whether the errors were distributed evenly across field staff?*

**Response**: We only categorised fields as receiving text or numeric inputs. The problematic text fields were evenly distributed across sites but with more problems in Nepal than Bangladesh than Malawi (see Table 1). Such differences across sites could be due to fieldworker abilities and experience (Figure 3). There was substantial reductions in errors post-retraining in Nepal and Bangladesh.

*A key issue with electronic data capture is the availability of internet access and the group has addressed the combination with offline collection and twice daily uploading. It would help to have clarity on whether there are country-level regulatory/legal issues with the data being hosted with the Oxford Vaccine Group?*

**Response**: Thank you, the study design was reviewed and approved by each country’s national research ethics committee as well as Oxford University. We provide approval numbers under “Ethics approval and consent to participate”.

*While this was not included in the scope of the paper, the authors’ comments on whether baseline census information data collection accuracy was comparable to data collected during follow-up or plans for future analysis would be helpful.*

**Response**: Thank you, we have added a sentence to capture your suggestion. Indeed we plan to re-evaluate accuracy following additional studies under STRATAA (Lines 348-249)

*Is the rationale for developing the new software tool clearly explained? Yes*

**Response**: Thank you.

*Is the description of the software tool technically sound? Yes*

**Response**: Thank you.

*Are sufficient details of the code, methods and analysis (if applicable) provided to allow replication of the software development and its use by others? Yes*

**Response**: Thank you.

*Is sufficient information provided to allow interpretation of the expected output datasets and any results generated using the tool? Yes*

**Response**: Thank you.

*Are the conclusions about the tool and its performance adequately supported by the findings presented in the article? Yes*

**Response**: Thank you.

# Peer review: Reviewer response for version 1

**DOI:** 10.21956/wellcomeopenres.17339.r39895

**Published:** 2020-09-03

**Authors:** Myron Levine, Michael Sikorski

**Affiliations:** 1 Center for Vaccine Development and Global Health, University of Maryland School of Medicine, Baltimore, MD, USA; 2 Center for Vaccine Development and Global Health, University of Maryland, Baltimore, MD, USA

The authors present a comprehensive custom electronic data capture system designed using open source tools and applied to a series of field censuses across three sites (one in Africa and two in Asia), targeting ~100,000 persons per site.

- The EDC system employs best-practices in data management, including quality control tools, automated data validation, and secure backups.
- The authors clearly describe the software data system and illustrate the flow of data using two effective figures. 
- All components of the software system are available in the public domain and clearly annotated.

The results are both credible and clear-cut with regards to data management. However, with respect to the ability to extrapolate this method to other sites, there is a basic assumption that high quality and trainable workers can be mobilized and dedicated to allocate 13-16 weeks on such an activity and that funding will be available for the personnel and transportation costs to utilize this EDC system in the field. We note that STRATAA represents a consortium of highly experienced sites with core funding and that each participating site was selected, in part, for their "paper-based studies of high participant volume and logistical complexity." 

- Expanded discussion of the limitations and external validity of this study would help; including a discussion of how comparator studies cited in the discussion were selected, when they were conducted, and whether they are comparable given the potential differences in technology.
- Major limitations to a successful demographic survey include the costs of the staff and the time/cost for transportation, stable WiFi, electricity, and GPS signals.

In summary: where adequate core funding, staffing, and transportation are readily available, the EDC system presented by the authors is likely to be readily adaptable as an upgrade to paper-based or out-moded electronic systems at a reasonable cost.

Are the conclusions about the tool and its performance adequately supported by the findings presented in the article?

Partly

Is the rationale for developing the new software tool clearly explained?

Yes

Is the description of the software tool technically sound?

Yes

Are sufficient details of the code, methods and analysis (if applicable) provided to allow replication of the software development and its use by others?

Yes

Is sufficient information provided to allow interpretation of the expected output datasets and any results generated using the tool?

Yes

Reviewer Expertise:

Field epidemiology and disease surveillance in LMICs; demographic surveillance; multi-site electronic data capture systems.

We confirm that we have read this submission and believe that we have an appropriate level of expertise to confirm that it is of an acceptable scientific standard, however we have significant reservations, as outlined above.

# Response

**Published:** 2020-11-23

**Authors:** Deus Thindwa

**Affiliations:** Malawi Liverpool Wellcome Trust, Malawi

*The authors present a comprehensive custom electronic data capture system designed using open source tools and applied to a series of field censuses across three sites (one in Africa and two in Asia), targeting ~100,000 persons per site.*

- *The EDC system employs best-practices in data management, including quality control tools, automated data validation, and secure backups.*
- *The authors clearly describe the software data system and illustrate the flow of data using two effective figures.*
- *All components of the software system are available in the public domain and clearly annotated.*

**Response**: Thank you.

*The results are both credible and clear-cut with regards to data management. However, with respect to the ability to extrapolate this method to other sites, there is a basic assumption that high quality and trainable workers can be mobilized and dedicated to allocate 13-16 weeks on such an activity and that funding will be available for the personnel and transportation costs to utilize this EDC system in the field. We note that STRATAA represents a consortium of highly experienced sites with core funding and that each participating site was selected, in part, for their "paper-based studies of high participant volume and logistical complexity."*

**Response**: We have added a sentence to acknowledge that our approach may not be a practicable scenario in some low-income settings. (Lines 328-331).

*Expanded discussion of the limitations and external validity of this study would help; including a discussion of how comparator studies cited in the discussion were selected, when they were conducted, and whether they are comparable given the potential differences in technology.*

**Response**: We have added a sentence to acknowledge limitations as per your suggestions. (Lines 304-307).

*Major limitations to a successful demographic survey include the costs of the staff and the time/cost for transportation, stable WiFi, electricity, and GPS signals.*

**Response**: We have added a sentence to capture the limitation of not being able to estimate other related costs. (Lines 364-366).

*In summary: where adequate core funding, staffing, and transportation are readily available, the EDC system presented by the authors is likely to be readily adaptable as an upgrade to paper-based or outmoded electronic systems at a reasonable cost.*

**Response**: Agree, we have added this to conclusions (Lines 375-376).

*Is the rationale for developing the new software tool clearly explained? Yes*

**Response**: Thank you.

*Is the description of the software tool technically sound? Yes*

**Response**: Thank you.

*Are sufficient details of the code, methods and analysis (if applicable) provided to allow replication of the software development and its use by others? Yes*

**Response**: Thank you.

*Is sufficient information provided to allow interpretation of the expected output datasets and any results generated using the tool? Yes*

**Response**: Thank you.

*Are the conclusions about the tool and its performance adequately supported by the findings presented in the article? Partly*

**Response**: We have expanded conclusions acknowledging what would be limitations of implementing the EDC (Lines 373-376).

# Peer review: Reviewer response for version 1

**DOI:** 10.21956/wellcomeopenres.17339.r39897

**Published:** 2020-09-02

**Authors:** Jillian S Gauld

**Affiliations:** 1 Institute for Disease Modeling, Seattle, WA, USA

This paper provides an excellent overview of a substantial electronic data capture system, implemented in three different locations.

 I appreciate the detailed tables and schematic figures, as well as the cost and timeline data being published. This study would be very helpful for other settings deciding on whether to implement such a system, and identify potential issues to be aware of.

 However, there is a narrative challenge throughout the paper. It is unclear after reading through the introduction, what the purpose of this particular paper was. Is it a summary of a novel tool? If so, what is novel about it as compared to other EDC’s? Or, if this is a descriptive study of a large scale implementation of an electronic system, what other large scale surveillance studies have failed to report this type of accuracy data, and how does this impact the quality of research? I think it would be helpful to the reader to more thoroughly ground the paper in a very clear introduction and purpose.

 Similarly in the discussion, I appreciated the comparison of the EDC’s costs and accuracy to other systems. However, there is only a limited discussion of what might be driving these differences. For example, are the different error rates driven by the rate of text-based data entry, or the type of disease being surveiled? For costs, are all of these studies geo-locating individuals? Investigating these differences further would very much strengthen the discussion and conclusions made.

 For Figure 3, I am not sure this is displayed correctly, or I may be misunderstanding what is being displayed. It appears that the post-retraining error is 1 minus the pre-retraining error for each field worker. Presumably some field workers may benefit less than others from the re-training?

 Some more minor comments:

**Methods**

- Paragraph 2 of the methods seems to be summarized at least partly in Figure 1, so would remove. 
- Some of the terms (preloading, looping) not obvious to a general audience, can you define?
- "All the other errors exposed by the external SQL queries were investigated thoroughly in the field before corrections could..." This section was a bit unclear - what are the other errors? Any non-GPS errors, or those that couldn't be auto-corrected through the SQL script?
- "The maximum number of visits to the household prior declaring the household vacant or errors permanently unresolved was twice." Would move this to results.
- The operation and implementation sections could be switched in order in the paper. 

**Discussion**

- The first time I see the 12 hour upload frequency is in the discussion unless I missed it, this should be in the methods somewhere.

Are the conclusions about the tool and its performance adequately supported by the findings presented in the article?

Partly

Is the rationale for developing the new software tool clearly explained?

No

Is the description of the software tool technically sound?

Yes

Are sufficient details of the code, methods and analysis (if applicable) provided to allow replication of the software development and its use by others?

Yes

Is sufficient information provided to allow interpretation of the expected output datasets and any results generated using the tool?

Yes

Reviewer Expertise:

Mathematical and statistical modeling of typhoid fever and other enteric diseases.

I confirm that I have read this submission and believe that I have an appropriate level of expertise to confirm that it is of an acceptable scientific standard, however I have significant reservations, as outlined above.

# Response

**Published:** 2020-11-23

**Authors:** Deus Thindwa

**Affiliations:** Malawi Liverpool Wellcome Trust, Malawi

*This paper provides an excellent overview of a substantial electronic data capture system, implemented in three different locations. I appreciate the detailed tables and schematic figures, as well as the cost and timeline data being published. This study would be very helpful for other settings deciding on whether to implement such a system, and identify potential issues to be aware of.*

**Response**: Thank you.

*However, there is a narrative challenge throughout the paper. It is unclear after reading through the introduction, what the purpose of this particular paper was. Is it a summary of a novel tool? If so, what is novel about it as compared to other EDC’s? Or, if this is a descriptive study of a large scale implementation of an electronic system, what other large scale surveillance studies have failed to report this type of accuracy data, and how does this impact the quality of research? I think it would be helpful to the reader to more thoroughly ground the paper in a very clear introduction and purpose.*

**Response**: The last paragraph of the introduction has been revised to make clear the purpose/rationale of developing the EDC (Lines 80-89).

*Similarly in the discussion, I appreciated the comparison of the EDC’s costs and accuracy to other systems. However, there is only a limited discussion of what might be driving these differences. For example, are the different error rates driven by the rate of text-based data entry, or the type of disease being surveyed? For costs, are all of these studies geo-locating individuals? Investigating these differences further would very much strengthen the discussion and conclusions made.*

**Response**: See our response to this to “Reviewer 1 point 13".

*For Figure 3, I am not sure this is displayed correctly, or I may be misunderstanding what is being displayed. It appears that the post-retraining error is 1 minus the pre-retraining error for each field worker. Presumably some field workers may benefit less than others from the re-training?*

**Response**: It interprets as the proportion of all errors committed per fieldworker e.g. if fieldworker 1 committed 10 errors pre-retraining and 5 errors post-retraining then 10/15 (67%) for pre-retraining and 5/15 (33%) for post-retraining are the % errors.

*Paragraph 2 of the methods seems to be summarized at least partly in Figure 1, so would remove.*

**Response**: In the spirit of keeping manuscript text independent from figures, we were previously advised by a reviewer to include the text in case some readers don’t have to check Figure 1.

*Some of the terms (preloading, looping) not obvious to a general audience, can you define?*

**Response**: Thank you, these terms have been defined on their first appearances. (Lines 120-122)

*"All the other errors exposed by the external SQL queries were investigated thoroughly in the field before corrections could..." This section was a bit unclear - what are the other errors? Any non-GPS errors, or those that couldn't be auto-corrected through the SQL script?*

**Response**: This sentence has been slightly revised by deleting prefix “All the other”. The sentence refers to errors listed under Lines 123-131

*"The maximum number of visits to the household prior declaring the household vacant or errors permanently unresolved was twice." Would move this to results.*

**Response**: Thank you, this has now been moved to results (Lines 250-251).

 The operation and implementation sections could be switched in order in the paper. 

**Response**: It is the Journal’s editorial advice to put them in the current order.

*The first time I see the 12 hour upload frequency is in the discussion unless I missed it, this should be in the methods somewhere*

**Response**: This has already been indicated in the Methods section. “Then data were collected in the field during the day and temporarily saved in the tablet’s memory. At the end of each day, tablets were returned to the base STRATAA data office and data were uploaded from the tablet’s memory to ODK Aggregate server via a secure wireless network technology.” (Lines 159-162)

*Is the rationale for developing the new software tool clearly explained? No*

**Response**: The last paragraph of the introduction has been revised to make clear the purpose/rationale of developing the EDC (Lines 80-89).

*Is the description of the software tool technically sound? Yes*

**Response**: Thank you.

*Are sufficient details of the code, methods and analysis (if applicable) provided to allow replication of the software development and its use by others? Yes*

**Response**: Thank you.

*Is sufficient information provided to allow interpretation of the expected output datasets and any results generated using the tool? Yes*

**Response**: Thank you.

*Are the conclusions about the tool and its performance adequately supported by the findings presented in the article? Partly*

**Response**: We have expanded conclusions acknowledging what would be limitations of implementing the EDC (Lines 373-376).

# Peer review: Reviewer response for version 1

**DOI:** 10.21956/wellcomeopenres.17339.r39774

**Published:** 2020-08-10

**Authors:** Sylvester Maleghemi

**Affiliations:** 1 World Health Organization, Port Harcourt, Nigeria

The article clearly demonstrates what was done, and can be replicated but does not clearly demonstrate what new information it adds to the scientific community, what was done different from previous ECDs, and leaves room for bias with facilities chosen for previous good performances. It also needs to speak more on the challenges and limitations especially in real time data updates to servers in low income settings where network remains an issue, collecting data and the assumption that dilapidated facilities etc. are justification for poor data is not proven and does not hold true.

 A 12 hour regular synchronization is really not feasible in our setting esp. Africa.

 Once these issues are addressed the paper should be considered for indexing.

 I have some specific comments that can be found in the PDF file here which need to be addressed.

Are the conclusions about the tool and its performance adequately supported by the findings presented in the article?

Partly

Is the rationale for developing the new software tool clearly explained?

Partly

Is the description of the software tool technically sound?

Yes

Are sufficient details of the code, methods and analysis (if applicable) provided to allow replication of the software development and its use by others?

Yes

Is sufficient information provided to allow interpretation of the expected output datasets and any results generated using the tool?

Yes

Reviewer Expertise:

My ares of research include Immunology, use of emobile technology for health, data management, clinical trials, research and International Health regulation.

I confirm that I have read this submission and believe that I have an appropriate level of expertise to confirm that it is of an acceptable scientific standard, however I have significant reservations, as outlined above.

# Response

**Published:** 2020-11-23

**Authors:** Deus Thindwa

**Affiliations:** Malawi Liverpool Wellcome Trust, Malawi

*The article clearly demonstrates what was done, and can be replicated but does not clearly demonstrate what new information it adds to the scientific community, what was done different from previous ECDs, and leaves room for bias with facilities chosen for previous good performances. It also needs to speak more on the challenges and limitations especially in real time data updates to servers in low income settings where network remains an issue, collecting data and the assumption that dilapidated facilities etc. are justification for poor data is not proven and does not hold true.*

**Response**: (a) We report a new application of an existing open source software platform. We show how this electronic system can be built and how it can be deployed to improve quality of huge volumes of data collection from across poor-resource settings, globally. We have amended the text accordingly. This manuscript is targeted at researchers who are considering adopting EDC. We have not formally compared our EDC with other systems, because this would require another large-scale census which was not feasible.

 (b) The selected study sites have considerable experience of paper-based data collection systems but no prior experience of using ODK at scale, particularly in high-density urban slums. The field workers using the EDC frequently had little prior experience of data collection. We have amended the text accordingly. (c) We agree with the reviewer that network interruptions could render EDC impractical, although network coverage and stability is improving in many resource-limited settings. To avoid this potential barrier to implementation, synchronisation between tablet and server was done at the data office, not in the field, and typically at night when connectivity was stable. d) Contrary to the Reviewer’s comments, we have presented the limitations of our system in the manuscript. These include errors from (i) using text fields, (ii) not retraining field workers for better performance or due to field worker steep learning curve, (iii) lack of knowledge and skills required to develop and manage the ODK-SQL apps, (iv) limited built-in functionality of ODK e.g. lack of detection of duplicate barcodes for individual IDs when scanned, (v) lack of ODK built-in GPS validations relative to map boundaries.

*A 12 hour regular synchronization is really not feasible in our setting esp. Africa.*

**Response**: On the contrary, one of the benefits of this system is that 12 hourly synchronisation was feasible. We describe a protocol whereby Android tablets were given out in the morning and returned in the late afternoon by field workers on a daily basis for collection and submission of data. We also provide datasets that have timestamps of data collection and submission for your review. (Lines 159-164)

*Is the rationale for developing the new software tool clearly explained?, Partly*

**Response**: The last paragraph of the introduction has been revised to make clear the purpose/rationale of developing the EDC (Lines 80-89).

*Is the description of the software tool technically sound?, Yes*

**Response**: Thank you.

*Are sufficient details of the code, methods and analysis (if applicable) provided to allow replication of the software development and its use by others?, Yes*

**Response**: Thank you.

*Is sufficient information provided to allow interpretation of the expected output datasets and any results generated using the tool?, Yes*

**Response**: Thank you.

*Are the conclusions about the tool and its performance adequately supported by the findings presented in the article?, Partly*

**Response**: We have expanded conclusions acknowledging what would be limitations of implementing the EDC (Lines 373-376).

*Competing Interests? No competing interests were disclosed.*

**Response**: The “Competing interests” statement was provided during submission in the online system. We have included it again in the revised manuscript:

*“Paper-based systems have the advantage that they provide a hard copy source document”. I agree but all advantages of paper can also be reflected in the ODK, rather focus on the benefits of the ODK, and its disadvantage here. The paper itself is not a comparism of paper based over emobile reporting* *(Lines 59-70)* *.*

**Response**: The inclusion of paper-based data collection drawbacks is to justify our choice for EDC (ODK). The major advantage of paper is in providing original copy of completed questionnaire. EDC may emulate that by providing an audit trail, but even an audit trail can be deleted leaving no evidence of changes. We list the advantages of EDC in the introduction, and discuss the disadvantages of EDC e.g. steep learning curve, poor form design which may result into errors during data collection.

*“In brief, the three sites, one in each country, were selected based on high known burden of enteric fever, differing epidemiological patterns and previous ability to deliver paper-based studies of high participant volume and logistical complexity.”* *The mannerism in which these sites were selected create a lot of room for bias, as the team purposively selected high performing facilities only that will deliver irrespective of challenges encountered especially in real life situation. Where in the article is STRATAA study designed discussed ??? (Lines 94-100)*

**Response**: The description of STRATAA study is given by reference number 13 - a peer-reviewed published paper by Darton TC et al. As discussed above, the selected sites had no prior experience of using EDC at very large scale.

*“Interestingly, the collection of such quality data is hindered by complexities of dilapidated health facilities, overcrowding, unstructured housing or slums, and illiteracy”.* *Cause and effect are not valid. The issue of data collection , quality etc and dilapidated health facilities "even good facilities can churn out poor data" same with the other reasons listed that affects the quality of data, you have not touched on major reasons for poor data in low and middle income countries such as simple things like even availability of the data tools, issues around data submission, from HFs to National database, quality of data due to poor supervision, DQAs etc. Please reword. (Lines 80-83)*

**Response**: We have amended the manuscript in line with the Reviewer’s suggestions (Lines 80-84).

*“Completeness of household demographics enumeration were 94.2%, 75.6% and 79.2% for Malawi, Nepal and Bangladesh, respectively, relative to background household count.” This is interesting, I would have thought this was an advantage of an interactive method of data collection, the rates between Malawi and the other 20 countries is greater than 20% and hopefully will be discussed in the discussions.*

**Response**: We have included a separate paragraph to explain variations in household data collection completeness across sites (Lines 309-312).

*“... and neglected tropic disease surveys (5.2%) in Ethiopia, the bloodstream infections study in Zanzibar (1.0%) and the tuberculosis program in India (4.2%)2,3,7,27. Moreover, our EDC data are more accurate in comparison to data reported from paper-based studies of maternal health (1.1%) and neglected tropical disease (6.2%) surveys in Ethiopia, bloodstream infections study in Zanzibar (7.0%), chronic disease research in South Africa (0.73%), and randomized controlled trial in Fiji (20.8%)”.* *Important that you have listed and compared your work with similar work and paper based, but you need to clearly state why this is more accurate, complete, as I am aware in most of the studies compared, training and retraining were done so what was done differently? (Lines 292-297)*

**Response**: We have already provided potential reasons for observed variations e.g. “The accuracy variations between EDCs are probably due to robustness of the EDC design in terms of error proofing. Robustness in the design is likely to depend on the limitations of software and hardware, and technical know-how of developers”. Also, we have added limitations in making such comparisons (Lines 303-307).

*“text fields of this eCRF generated more errors than numeric fields, and suggest that such errors could be prevented in eCRF designs by minimizing the use of text fields through coding of text responses or leaving out insignificant text responses completely.”* *Using a Drop box with selected comments is usually the standard whereby ticks become application, the idea of collecting data is for result, however it will be good to explain what can of information was been sourced from the field text in the beginning and its relevance. (Lines 297-300)*

**Response**: Figure 1 provides a flowchart with different data types for different fields and indicates what kind of information was sourced as text. In the Methods section, we state all fields of the eCRF and their relevance. (Lines 104-116).

*“Data were synchronized from client to server at a later point in time at the base STRATAA data office where connectivity was possible. This approach has also been recommended by others.”* *This introduces a limitation, that should be discussed, in some countries the data office may not assessed in days, as it seems the households targeted ensured that the surveyors could reach the offices daily and this is not a practicable scenario in most low income countries. I am aware also that most countries have learnt to rely on GSM networks to upload this data to mitigate this and ensure that the data collected included pictures are compressed on the ODK to ensure success of its transmission to the server. (Lines 316-318)*

**Response**: Thank you, additional statement to support this as a limitation in some low-income study sites far from the data office has been added. (Lines 328-331).

*“In conclusion, we have designed an EDC which has been implemented in three typhoid endemic sites to collect large volume of accurate.” I agree with the conclusion but I am of the opinion that the study itself is oversimplified to just 3 things*

1. *How data was collected using emobile technology along with its costs, training etc*
2. *How it compares with other similar data collection mechanism ie paper and other studies*
3. *The most interesting is how the data itself was managed along with the SQL *

**Response**: Thank you.

*What it has failed to bring out is*

*Understanding the potential impact of apps as part of a comprehensive integrated approach required for data collection.*

**Response**: We have presented the impact of the EDC as being capable to manage collection and processing of huge volumes of high quality data over very short time periods across different settings at a moderate cost. Therefore we maintain that the tool is an essential part of the integrated approach required for data collection (text amended).

*The limitations have not been properly addressed as it seems that all is easy in real life data collection*

**Response** *: *The Reviewer may have missed the limitations section of the manuscript. We have presented limitations in terms of (i) more errors from using text fields,(ii) a need to retrain field workers for better performance or due to steep learning curve, (iii) the knowledge and skills required to develop and manage the ODK-SQL apps, (iv) limited built-in functionality of ODK like lack of detection of duplicate barcodes for individual IDs when scanned, (v) accuracy of GPS can only be controlled with external Google Earth software and SQL queries, and not ODK alone.

*What is new as EDC has been and is continuously been used across different health programmes.*

**Response** *: *We report a new application of an existing open source software platform. We show how the system can be built and deployed to improve quality of huge volumes of data collection from across poor-resource settings. This paper is targeted at researchers who are considering adopting EDC.
